# The assessment of caregiver self-efficacy in a virtual eating disorder setting

**DOI:** 10.1186/s40337-023-00869-x

**Published:** 2023-09-22

**Authors:** Nickolas M. Jones, Jessica H. Baker, Bek Urban, David Freestone, Angela Celio Doyle, Cara Bohon, Dori M. Steinberg

**Affiliations:** 1 Equip Health, Inc., CA Carlsbad, USA; 2 University of California, Irvine, CA USA; 3 Stanford University, Stanford, CA USA; 4 Duke University, Durham, NC USA; 5 Carlsbad, USA

**Keywords:** Eating disorder, Family therapy, Anorexia nervosa, Self efficacy

## Abstract

**Background:**

Caregiver self-efficacy is thought to be a key component for successful family-based treatment (FBT) for individuals with eating disorders. As such, interventions aimed at enhancing caregiver self-efficacy, often measured via the Parents Versus Anorexia scale, have been a focal point of FBT literature. However, studies looking at the relationship between caregiver self-efficacy and treatment outcomes have been mixed. We aimed to better understand the influence of caregiver self-efficacy on eating disorder treatment outcomes during FBT.

**Methods:**

Caregiver self-efficacy was measured using the Parents Versus Eating Disorders (PVED) scale, an adapted version of the Parents Versus Anorexia scale, in a sample of 1051 patients with an eating disorder and 1528 caregivers (patients can have more than one caregiver) receiving virtual FBT. Across two multilevel models, we tested how caregiver self-efficacy changed over time and its association with changes in eating disorder symptoms and weight over the first 16 weeks of treatment.

**Results:**

Over treatment, PVED scores increased (*b* = 0.79, *SE* = 0.04, CI [0.72, 0.86]) and starting PVED scores were predictive of improved eating disorder symptoms (*b* = − 0.73, *SE* = 0.22, CI [− 1.15, − 0.30]), but not weight (*b* = − 0.96, *SE* = 0.59, CI [− 2.10, 0.19]). We also found that PVED change-from-baseline scores were predictive of weight (*b* = − 0.48, *SE* = 0.03, CI [− 0.53, − 0.43]) such that patient weight was lower when caregiver reports of PVED were higher. Likewise, the association between caregiver change in PVED scores and weight varied as a function of treatment time (*b* = 0.27, *SE* = 0.01, CI [0.24, 0.29]). Results were consistent when isolating patients with anorexia nervosa.

**Conclusions:**

Caregiver self-efficacy during FBT improved over time but was not robustly associated with treatment outcomes. This may, in part, be due to psychometric properties of the PVED scale. We describe these issues and illustrate the need for development of a new measure of self-efficacy for caregivers supporting their loved ones through eating disorder treatment.

## Abstract (plain language summary)

In family-based treatment (FBT) for eating disorders, caregivers play a central role in helping their child recover. Specifically, a caregiver’s self-efficacy, or their confidence in their ability to cope with the challenges their child faces during treatment, is believed to be a key to successful treatment outcomes. However, research on the impact caregiver self-efficacy has on patient treatment outcomes is mixed. Using the Parent vs. Eating Disorder (PVED) scale, we looked at the association between caregiver self-efficacy and eating disorder symptoms and weight changes over time in a sample of 1051 patients receiving virtual FBT. Caregiver self-efficacy had little to no significant impact on treatment outcomes. We offer explanations about why our findings challenge existing research that supports the association between caregiver self-efficacy and outcomes. Namely, the PVED scale appears to measure caregiver perceptions of their role in the treatment process (and not self-efficacy). We believe a new measurement tool must be developed and tested for the field to move forward in its understanding of the role caregiver self-efficacy plays in FBT.

## Introduction

Family-based treatment (FBT) is considered the gold-standard treatment for children and adolescents with an eating disorder. In randomized controlled trials, FBT has resulted in better remission outcomes than comparison treatments for adolescents with anorexia nervosa, bulimia nervosa, and, in a pilot study, avoidant restrictive food intake disorder (ARFID) [1–5]. Although FBT is a promising treatment, professionals still have limited knowledge about what predicts a successful outcome for children and adolescents receiving FBT. This is a critical concern given that only approximately 50% of youth fully recover after a course of FBT [6]. Family members are also an integral component of FBT, and clinicians often view parental empowerment as the most important part of FBT [7]. Indeed, caregiver or parental empowerment to bring about their child’s recovery is one of the core tenants of FBT [8]. Therefore, it is also critical to understand parental factors associated with effective FBT, which has generally been overlooked to date.

In FBT, parents and/or other caregivers are included into the treatment team along with a trained psychologist or therapist, medical provider, and a dietitian [9]. It is the caregivers’ responsibility to guide and supervise at-home aspects of treatment during the early stages of care. For example, a primary role of caregivers is to supervise and monitor meals and eating disorder behaviors, which supports the child’s recovery [9]. FBT can also address the family burden of the illness by providing coping strategies to manage challenges related to their child’s eating disorder [10]. The mealtime structure FBT provides, a pragmatic approach to symptom reduction, as well as support from the FBT therapist, helps caregivers increase their ability to cope with their child’s eating disorder and improve familial relationships [11]. As progress is made in treatment, patients develop skills that enable them to take on more age-appropriate responsibilities and become increasingly independent.

Focus groups with parents involved in FBT to treat their child’s eating disorder have found parents desire extra emotional support from treatment teams and eating disorder specific education that bolsters empowerment [12]. In the context of FBT, parental empowerment has been defined as building parental confidence to take on the role of primary agent of change in their child’s recovery process, as building confidence allows caregivers to stand firm against their child’s eating disorder [8]. A goal of parental empowerment, then, is to improve parent or caregiver self-efficacy in filling their important role in FBT, which may address caregiver concerns and improve treatment outcomes [13].

Self-efficacy refers to one’s perceived ability or confidence to execute a specific behavior across a variety of situations [14]. Within FBT, parental self-efficacy is the caregivers’ perceived ability to help their child recover from the eating disorder [13, 15]. Indeed, some studies have directly linked parental self-efficacy with better treatment outcomes [i.e., fewer eating disorder symptoms [13]] while others have specifically tied parental self-efficacy to greater weight gain [15, 16]. Such findings tying parental self-efficacy to positive treatment outcomes has resulted in increased attention to finding ways of supplementing FBT to bolster parental self-efficacy [17–19]. Multiple interventions to supplement FBT with a goal of improving parental self-efficacy have been studied, including providing structured parental psychoeducation, parental support groups, multi-family groups, and multifamily meals, among others [17–19]. Previous interventions designed to increase self-efficacy among parents in FBT have reported increased self-efficacy, yet parental self-efficacy was not associated with patient treatment outcomes [18, 20].

Other findings further complicate the understanding of parental self-efficacy as a predictor of treatment outcomes and suggest that the type of caregiver or parent (e.g., mother vs. father) may moderate the role of caregiver self-efficacy in treatment outcomes [16]. Some authors suggested that parental self-efficacy may influence treatment outcomes in FBT, yet not all studies support this assertion [21]. Hamadi and Holliday [21] discuss inconsistent findings in their review of factors that impact outcomes of adolescents receiving FBT treatment. The authors argue that, although parental self-efficacy has largely been accepted as a factor impacting treatment outcomes, the empirical literature is mixed (see [20–23], for further discussion and noted inconsistencies). Further, most studies evaluating parental self-efficacy to date have had a limited sample size, impacting statistical power, which in turn hinders the ability to detect reliable associations and test for moderators

Taken together, the aim of this study is to address several gaps in the literature to date. First, given the importance of caregivers in FBT, we evaluate the impact of parental factors on child treatment outcome. Second, we harness a large sample of young adults receiving FBT for an eating disorder. Specifically, we evaluate whether caregiver self-efficacy is associated with patient outcomes (i.e., eating disorder symptoms, weight) within the first 16-weeks of treatment.

## Method

### Participants and treatment overview

The sample for this study included patients (N = 1051) and their caregivers self-selecting for eating disorder treatment at a national, virtually-delivered treatment program serving patients ages 6–24. Patients were either actively engaged in treatment or discharged from treatment with a diagnosis of anorexia nervosa, bulimia nervosa, binge-eating disorder, or other specified feeding and eating disorder, between the end of December 2020 and November 2022. Patients with ARFID were not included in this study.

The virtual treatment program delivers an enhanced version of FBT (FBT+) treatment of eating disorders. In FBT, a primary role of caregivers is to supervise and monitor meals and eating disorder behaviors, which support the child’s recovery. In traditional FBT, treatment is led by a therapist and the team may include a registered dietitian and physician [9]. The FBT+ approach includes a registered dietitian and physician as a standard member of the multidisciplinary treatment as well as a peer mentor and family mentor who have lived experience with an eating disorder. The peer mentor has recovered from an eating disorder and provides patients with a source of hope and motivation towards recovery. The family mentor has supported a loved one in recovery from an eating disorder and shares examples of skills and strategies with caregivers. If a family/patient chooses to not engage with a mentor, this is discussed with other members of the team in sessions but not forced; however, the mentor remains an integral member of the patients treatment team providing critical lived experience to other team members.

Our enhanced version of FBT is deployed in a way that fits the needs and specific presentation of the individual patient and family. Cadence of sessions is determined by the treatment team and as treatment progresses, tapering of sessions also occurs at the consensus of the treatment team and family generally occurring around the transition to Phase 3. All members of the care team meet with the family virtually through a Health Insurance Portability and Accountability Act (HIPAA) compliant platform approximately once per week. Following the traditional FBT model, FBT+ sessions are scheduled for 50-min sessions. More details about the FBT+ treatment approach and its effectiveness can be found elsewhere [22].

### Measures

As a part of routine protocols, patients and caregivers completed empirically validated surveys during the course of treatment through a HIPAA compliant electronic health record.

### Eating disorder symptoms

The Eating Disorder Examination Questionnaire Short Form (EDE-QS) was used to assess eating disorder severity [23]. This 12-item measure assesses eating psychopathology over the past seven days. Each item is rated on a four-point Likert-type scale ranging from 0 (no days) to 3 (six to seven days). Example items include “Have you had a definite fear that you might gain weight?” and “Have you tried to control your weight or shape by making yourself sick (vomit) or taking laxatives?” Scores can range from 0 to 36, with higher scores indicating more severe eating psychopathology. Scale reliability was estimated at Cronbach’s *ɑ* = 0.90 and EDE-QS measurements were collected weekly during treatment.

### Weight

Patient weight was measured at home by a caregiver who was trained in weight monitoring procedures during orientation to FBT+. Weight was measured by trained caregivers twice weekly with the patient wearing minimal clothing, after voiding, and prior to food or beverage consumption. The electronic health record delivered an automated prompt to the caregiver to enter the weight via text or directly in the electronic health record.

### Caregiver self-efficacy

The Parents Versus Eating Disorders (PVED) scale was completed by caregivers at admission and monthly thereafter. If patients have more than one primary caregiver involved in treatment, all caregivers are asked to complete the measure. The PVED is an adapted version of the Parents Versus Anorexia scale [24], which replaces the term “anorexia nervosa” with “eating disorder” to allow for use across various eating disorders. Previous studies exploring caregiver self-efficacy also use this minor change in wording to allow the scale to be applicable to experiences with any eating disorder [13, 17, 18, 25]. The Parents Versus Anorexia scale was developed for research of the Maudsley model of family therapy and designed to evaluate a caregiver’s ability to take charge of the eating disorder in the home [24].

The PVED scale contains seven items measured on a five-point Likert-type scale ranging from zero (strongly disagree) to five (strongly agree). Example items include, “I feel equipped with specific practical strategies for the task of bringing about the complete recovery of my child in the home setting,” “It is more my responsibility than my child’s to bring him/her to a healthy weight” and “I don’t have the knowledge to take a leadership role when it comes to achieving a total victory over the eating disorder.” Total scores can range from seven to 35 with higher scores indicating greater perceived caregiver efficacy. Scale reliability was estimated at Cronbach’s *ɑ* = 0.61 in our sample, which is slightly lower than reported internal consistency during scale development [24].

### Analytic strategy

We evaluated the impact of PVED scores on patient treatment outcomes during the first 16 weeks of FBT+. This time frame was chosen because it generally aligns with the end of treatment time frame in FBT randomized clinical trials [1]. We specified a series of multilevel models, as indicated below. The first model estimated PVED scores over time for caregivers (i.e., mothers and fathers). The model took the following form: PVED_w,j_ ~ β_0_ + β_0j_ + β_1_log(*w*) + β_1j_log(*w*) + β_2_carergiver + β_3_[caregiver × log(*w*)]; where *w* represents the treatment week (we take the log because treatment progresses logarithmically over time [22]) and j indexes the patient (so that β_0j_ and β_1j_ represent random intercepts and slopes). The random effects account for different patient-level starting points and PVED change trajectories over time. The *caregiver* term represents the member of the patients FBT + support group that provided the PVED score in week *w*. This model outperformed a model that did not account for caregiver type (difference in expected log predictive density: 107.6 ± 14.9; difference in WAIC: 110.3 ± 15.0).

We next specified two multilevel models to estimate how eating disorder symptoms and weight (our primary outcomes) improved week-by-week through treatment, emphasizing the estimates of the association between PVED scores and these outcome trajectories. In weeks with more than one survey measurement, we averaged the measurement to obtain a single weekly value. Because we treat carers as a unit supporting the patient, we also averaged over caregiver PVED scores if they occurred in the same week.

Both models took the following form: y_wj_ ~ β_0_ + β_0j_ + β_1_log(*w*) + β_1j_log(w) + β_2_PVED_0_ + β_3_PVED_Δ_ + β_4_[log(w) × PVED_0_] + β_5_[log(w) × PVED_Δ_]; where *w* represents the treatment week, j indexes the patient (so that β_0j_ and β_1j_ represent random intercepts and slopes), PVED_0_ represents the PVED score at week 0, and PVED_Δ_ represents the PVED change-from-baseline score in any given week *w*. The fixed effects portion of each model also included an interaction between the two PVED terms and treatment week.

This analytic approach allowed us to examine: (a) overall change in the treatment outcome over time, (b) separate the effects of the initial PVED score (a largely between subject variable) and the change in PVED over time (a largely within-patient variable), and (c) whether outcomes over treatment time varied as a function of PVED scores. We also included random intercepts and slopes in the model (β_0,j_ and β_1,j_log(w)) to allow for individual differences in initial outcome values and individual trajectories through treatment. Given that the EDE-QS is an ordinal measure, we used an cumulative logistic link function (i.e., ordinal regression) to fit the model to these data [26]. Weight is a positive and an approximately continuous measurement, so it was fit with a standard gaussian link function.

The sample size for each model below differs slightly due to missing data. Even though both EDE-QS and weights were collected each week, patients/caregivers sometimes failed to complete the measurement. In some cases, carers failed to fill out the initial PVED survey, so we could not estimate PVED change scores for those families. Patients excluded from the analyses because of missing data did not differ demographically to those included.

We used Hamiltonian Monte Carlo to generate samples from the posterior distribution of the joint parameter distribution. This approach requires setting priors on the parameters to regularize the fits. We chose priors that fit the domain of the parameter, but otherwise kept the priors conservative. For example, our prior on log(*w*) (i.e., treatment week) for the EDE-QS outcomes model was (0, 2) even though we have prior data to show that we expect this parameter estimate to be negative, and around − 2. It was set at (0, 6) in the weight outcomes model, even though we have prior data suggesting it should be closer to 3 or 4. Priors for all fixed effects covariates in the model were normally distributed priors centered around zero (null effect) but wide enough to capture larger effects. This results in a conservative test because the data has to overcome a higher prior probability density around the null hypothesis. The priors for the intercepts were chosen similarly: centered at zero for the EDE-QS ordinal model, and centered at 110 lbs for the weight model [based on [22]]. The priors on the random effects intercept and slope were half-(0, 4), to allow for large individual differences.

All analyses were conducted with R [27] using the tidyverse version 2.0 [28]. Fitting was done using the brms package version ​​2.18.0 [29], a wrapper around the Stan probabilistic programming language [30]. Model coefficients are presented with their corresponding 95% credible intervals. We consider a finding reliable if the credible intervals do not contain zero as a plausible value for the parameter. If zero is contained within the credible intervals, we consider the null hypothesis plausible. Project management was done using the targets package version v1.0.0 [31].

## Results

### Participants

Patients were on average 15 years-old (*M* = 15, *SD* = 2.43); patients were predominantly cisgender girls/women (*n* = 862; 82.0%) and boys/men (*n* = 85; 8.0%). Approximately 9.0% of patients (*n* = 95) did not identify as cisgender and gender was unknown for nine patients. In all, 90.6% (*n* = 953) patients were diagnosed with anorexia nervosa, 3.4% (*n* = 36) with an unspecified eating or feeding disorder, 2.7% (*n* = 29) diagnosed with bulimia nervosa, and 2.0% (*n* = 22) binge-eating disorder. In all, 46.0% of the patient sample completed at least 16 weeks of treatment. Throughout the treatment period under study, 174 patients were discharged before 16 weeks, and 131 patients had fewer than 16 weeks but were still active in treatment. The remainder of the sample remained active and completed 16 weeks of treatment. All patients with valid data were retained for further analysis, regardless of discharge status.

**Fig. 1:**
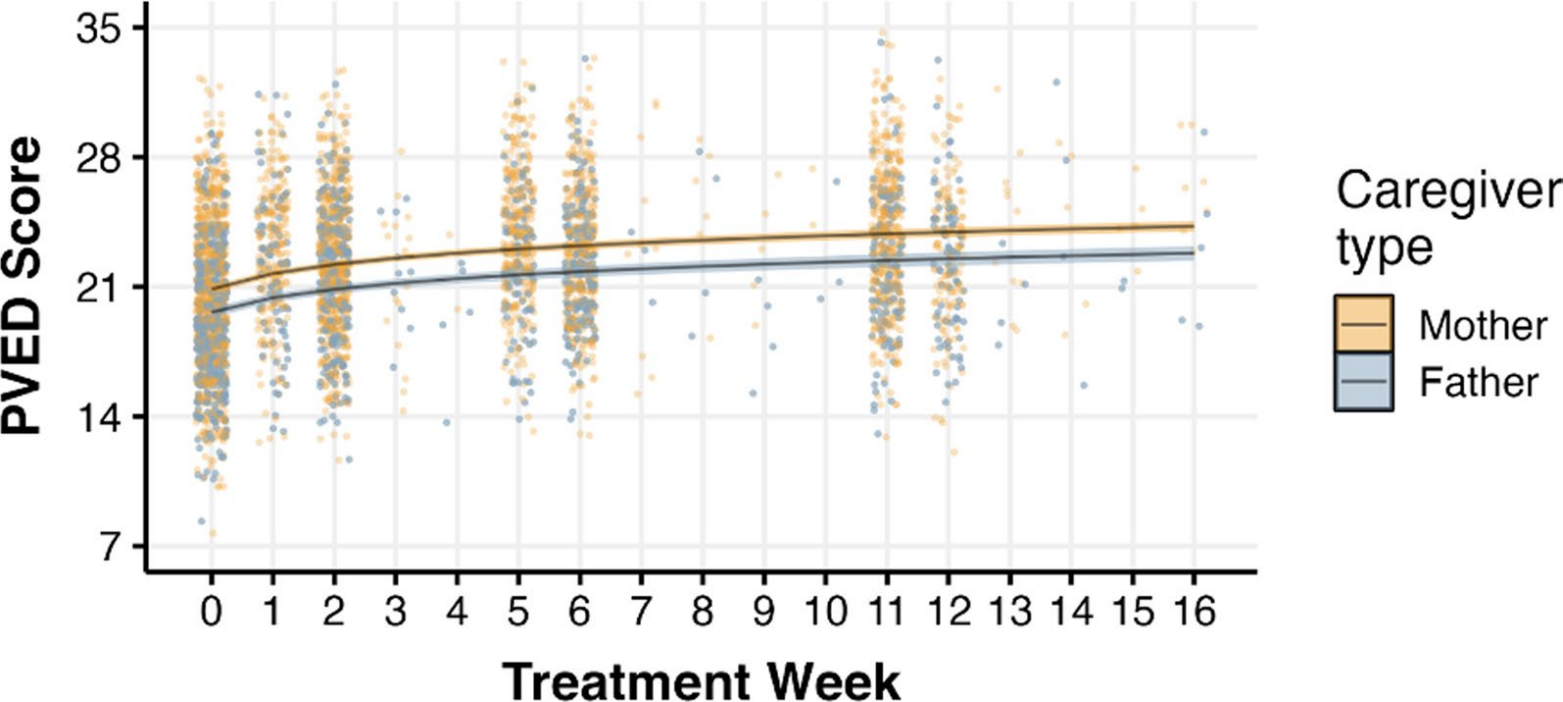
Predicted PVED scores over treatment time by caregiver type, and the corresponding weekly distribution of raw scores for each caregiver type

The sample included PVED ratings from 1,528 caregivers. Just over half of patients (53%) had at least two carers complete the PVED measure throughout treatment. Of these caregivers, 62.1% (*n* = 949) self-identified as mothers (including step, adoptive, and foster mothers), 36.9% (*n* = 565) self-identified as fathers, and the remainder reported other relationships with the patient (e.g., aunt, grandparent, significant other). At the onset of treatment, mothers reported an average PVED score of 20.58 (*SD* = 3.94), fathers reported 19.50 (*SD* = 3.56), and other caregiver types reported 20.00 (*SD* = 2.48); mothers reported slightly higher PVED scores than fathers (M_difference_ = 1.08, *t*(1512) = 5.36, *p* < .001) but this may not be a clinically meaningful difference. Likewise, there was no difference in the rate of PVED change over treatment by caregiver type (*b* = − 0.06, *SE* = 0.07, CI [− 0.19, 0.07]). On average, across all caregivers, PVED scores significantly increased over treatment time (*b* = 0.79, *SE* = 0.04, CI [0.72, 0.86]; see Fig. 1).

### Eating disorder symptoms

This model was fit using 10,596 observations across 895 patients. Eating disorder symptoms decreased over 16 weeks of treatment (*b* = − 1.87, *SE* = 0.10, CI [− 2.06, − 1.67]). The model term for starting PVED score estimated the between-patient difference in outcomes for a patient whose caregivers had higher initial PVED scores relative to the average score in our sample. Starting PVED scores were predictive of improved [decreased] eating disorder symptoms (*b* = − 0.73, *SE* = 0.22, CI [− 1.15, − 0.30]) such that at the onset of treatment, patients reported fewer eating disorder symptoms when their caregivers reported elevated PVED scores. Starting PVED scores were not moderated by treatment time (*b* = − 0.02, *SE* = 0.10, CI [− 0.22, 0.18]).

The term for PVED change-from-baseline scores estimated the extent to which change in PVED scores over time was associated with change in outcomes. PVED change scores were not predictive of eating disorder symptoms (*b* = 0.03, *SE* = 0.03, CI [− 0.03, 0.09]) and likewise did not moderate the association between treatment time and EDE-QS scores (*b* = .− 0.02, *SE* = 0.02, CI [− 0.05, 0.02]).

### Weight

This model was fit using 15,873 observations across 863 patients. Weight increased over 16 weeks of treatment (*b* = 3.47, *SE* = 0.13, CI [3.20, 3.72]). Starting PVED scores were not significantly associated with weight (*b* = − 0.96, *SE* = 0.59, CI [− 2.10, 0.19]). Likewise, the association between treatment time and weight did not depend on starting PVED scores (*b* = 0.16, *SE* = 0.12, CI [− 0.08, 0.39]).

**Fig. 2:**
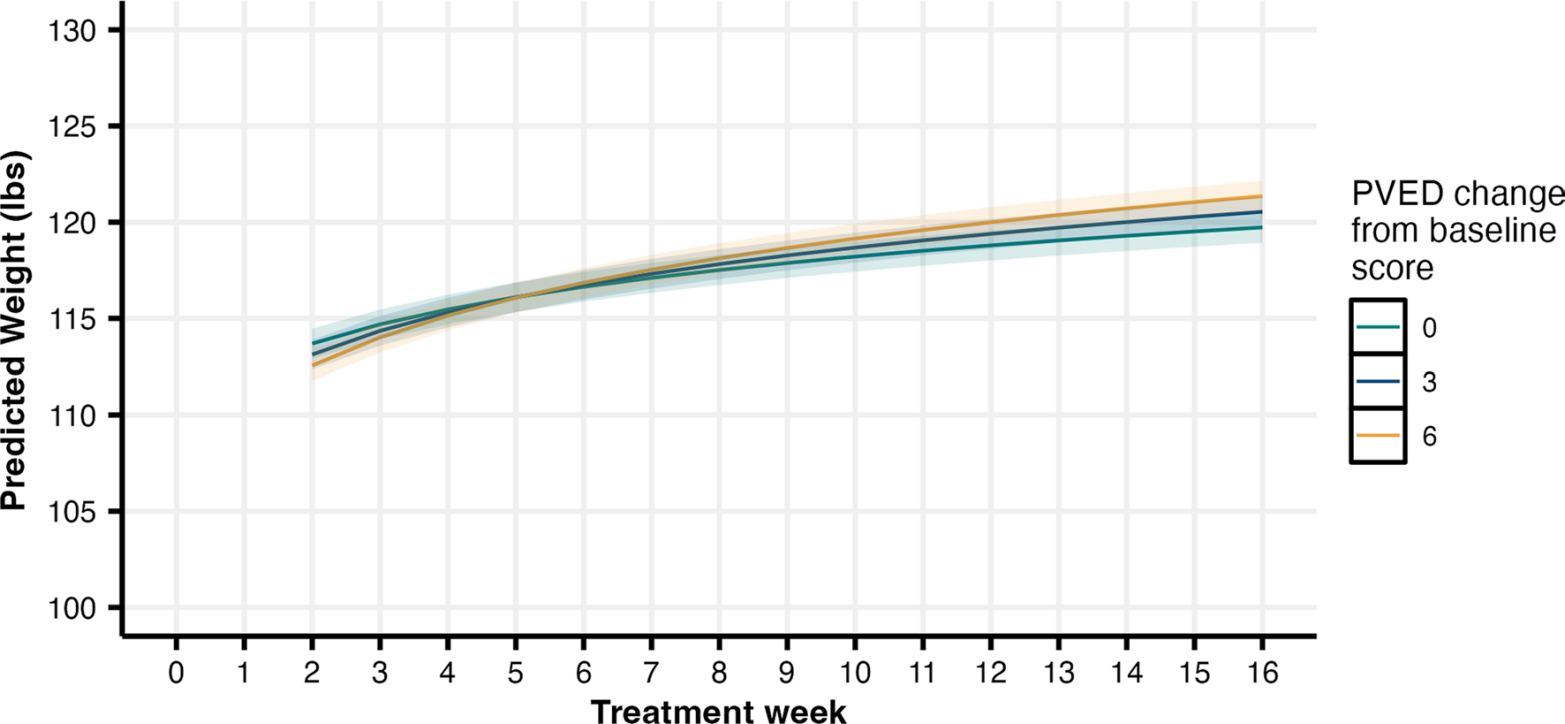
Predicted weight throughout treatment, broken out by recent PVED change

PVED change-from-baseline scores were predictive of weight (b = −.48, SE = .03, CI [−.53, −.43]) such that patient weight was lower when caregiver reports of PVED were higher. Likewise, the association between caregiver change in PVED scores and weight varied as a function of time in treatment (b = .27, SE = .01, CI [.24, .29]). Early in treatment (e.g., the first month), how a carer’s PVED score changed had little bearing on weight. However, by 16 weeks of treatment, greater increases in PVED scores were associated with higher weight relative to similar PVED gains earlier in treatment (see Fig. 2).

### Post-hoc analyses

Given that most studies of parental self-efficacy focus on individuals with anorexia nervosa, we also repeated the above analyses for eating disorder symptoms and weight including only patients with anorexia nervosa. The pattern of results did not change with only this subsample included in each analysis. Finally, we tested whether caregiver type (i.e., mother and father) moderated the relationship between PVED scores and each outcome. Across all models, caregiver type was not a significant moderator (EDE-QS: *b* = 0.01, *SE* = 0.01, CI [− 0.02, 0.03]; weight: *b* = − 0.01, *SE* = 0.01, CI [− 0.02, 0.02]).

## Discussion

Caregiver self-efficacy has been proposed to significantly impact patient eating disorder treatment outcomes during FBT [24], given that caregivers are an integral component to the treatment team [6]. However, the empirical evidence supporting this assertion is mixed [16]. In our large sample of patients receiving an enhanced version of FBT (FBT+), we observed a small or null association between caregiver self-efficacy and treatment outcomes. Further, although caregiver self-efficacy statistically moderated the association between time in treatment and weight change, the magnitude of this moderation was small and may not be clinically meaningful.

There are possible explanations for why we found small or null associations between caregiver self-efficacy and eating disorder treatment outcomes, as well as why the research to date evaluating this has been mixed. First, the Parent Versus Anorexia questionnaire, which we modified to create the PVED, is one of the most commonly used measures to examine parental self-efficacy within the context of FBT. Yet, the internal consistency of this measure in our sample was low, and below the threshold generally considered adequate [32]. Low internal consistency aligns with other published and unpublished findings of the internal consistency of the PVA, indicating reliability is consistently low [22, 33]. Inconsistent reliability of the PVA/PVED is likely one important factor as to why results across studies in the literature are mixed. An unreliable measure is going to produce inconsistent results.

A review of the literature using the PVA scale reveals that the intended use of the measure may have also changed over time. As described by Rhodes and colleagues (2005), the PVA was developed as a measure of *parental efficacy*, defined by developers as “the ability of a parent to adopt a primary role in taking charge of the anorexia in the home setting for the purpose of bringing about the recovery of their child” [26]. Over the past decade, the description of this scale has transitioned to a measure of parental *self-efficacy* [16, 21, 33, 34]. Though nuanced, *efficacy* and *self-efficacy* are not entirely the same. Decades of research posits that self-efficacy involves a person’s confidence in their ability to exert control over their motivation, behavior, and environment across different contexts [14]. Thus, standard measures of self-efficacy (across domains) frame items in a way that captures facets of one’s confidence and belief in their ability to do a task or cope with challenge.

In contrast, most PVA items query parents/caregivers’ perceptions of their role in FBT and how well parents/caregivers believe in the treatment process itself. For example, “I feel equipped with specific practical strategies for the task of bringing about the complete recovery of my child in the home setting” and “It is more my responsibility than my child’s to bring him/her to a healthy weight.” The items make sense given the PVA was designed to parallel the six core principles of the Maudsley model [24] and be consistent with the theoretical constructs of Maudsley. Taken together, the standard scale used in the literature to measure parental self-efficacy in FBT may not accurately represent how it was developed. A measure of parental self-efficacy during FBT should measure a caregiver’s perceived confidence and ability to bring their child through eating disorder treatment. In contrast, the PVA (and the adapted version used in the current study; i.e., PVED) captures parental perceptions of their role in treatment and agreement with the FBT theoretical model–namely parental “buy-in” to FBT. Based on this, we can conclude that improving parental buy-in to FBT may not be enough to improve patient treatment outcomes.

Second, it is possible that PVED scores do not operate as we predicted. Here, we surmised that it operates consistently over time and thus we would find a systematic relationship with outcomes across time in treatment. However, PVED scores may function differently at different points during treatment. For example, others have shown an association between caregiver self-efficacy and treatment outcomes at 3 and 6 months after treatment [13]. Insofar as the impact of caregiver self-efficacy may vary with time, examining an overall association may not be the best fit to do the data. Other moderators may also exist such as family structure or disease burden. For example, nonintact families may experience greater illness burden [[35]; however see [36]] and as such, it is possible parental self-efficacy is more important for families reporting greater burden. Finally, our FBT + program provides additional support for parents not part of traditional FBT (e.g., family mentors). This consistent and enhanced family support, specifically the caregiver/patient level impact of mentors, could impact findings in comparison to previous studies.

We take the transition of the conceptualization of the PVA scale over the past decade along with measurement challenges to be limitations of the current (and previous) work and potential factors that have contributed to the pattern of mixed findings in the literature. Although the PVA is widely used as a measure of caregiver self-efficacy, it was developed to measure a different, yet related, construct. It is imperative that future research construct and rigorously test a measure of caregiver self-efficacy that can be useful for answering questions about its role in the FBT process. Our study also has several strengths. We used a large clinical sample to study caregivers’ perception of FBT and its association with eating disorder symptoms and weight. Moreover, we analyzed our data using advanced longitudinal modeling that takes into account the ordinal nature of severity of eating disorder symptoms data and provides estimates of the associations that are fit using prior knowledge (i.e., a Bayseian framework). Our analyses were also conducted with granular data (weekly outcomes measurements), which has been limitedly done previously.

## Conclusions

Caregiver self-efficacy may be an important factor that bolsters desirable outcomes among patients in FBT for an eating disorder. Increasing a caregivers’ belief in their ability to help the adolescent patient, as well as their confidence in providing needed support, makes logical sense as a factor that would improve treatment outcomes. However, in order to fully understand whether this is the case, a suitable measure of caregiver self-efficacy is needed. Understanding and measuring parental self-efficacy as a construct well grounded in self-efficacy research will open the door to new avenues of eating disorder research. Indeed, general self-efficacy measures have been used by eating disorder researchers in past studies [15, 16, 37] and may be an ideal starting point to develop a measure specific to caregivers of a child with an eating disorder. Furthermore, a new measure of parental self-efficacy in the eating disorder field will allow us to study the basic association between this construct and treatment outcomes; and over time include moderators, mediators, and nonlinear relationships. Separating self-efficacy from the other possible construct(s) that PVED measures allows us to better measure both the effects of self-efficacy and those other constructs (e.g., parental buy-in, or caregiver attitudes toward their role in treatment). Given the percentage of adolescents who do not fully recover from an eating disorder after a course of FBT, further understanding the importance of family factors in treatment may increase the likelihood of full recovery.

## Data Availability

The data that support the findings of this study are derived from patient medical record data. The data are not publicly available and individual data cannot be shared due to privacy restrictions.

## Abbreviations

FBT: Family-based treatment
PVA: Parent Versus Anorexia
PVED: Parent’s versus eating disorders
EDE-QS: Eating Disorder Examination Questionnaire Short Form
